# Conflicts hinder research into animal movements

**DOI:** 10.1007/s13280-026-02395-x

**Published:** 2026-04-13

**Authors:** Víctor Martín-Vélez, Joan Navarro, Isabel Afán, Tomas Montalvo, Andy J. Green

**Affiliations:** 1 Institut de Ciències del Mar (ICM), CSIC, Barcelona, Spain; 2 Agencia de Salut Publica de Barcelona, Barcelona, Spain; 3 Estación Biológica de Doñana (EBD), CSIC, Seville, Spain

**Keywords:** Diseases, GPS tracking, Gulls, Migration, One health, Spoofing

## Abstract

Satellite tracking has revolutionized our understanding of animal migration, yet its reliability increasingly depends on the geopolitical stability of the regions frequented by wildlife. Here, we show that military-induced interference with global navigation satellite systems (GNSS) during ongoing conflicts in Eastern Europe has severely compromised the accuracy of global positioning systems (GPS)-based tracking data for black-headed gulls (*Chroicocephalus ridibundus*). In 2024–2025, GPS trajectories revealed erratic, low-quality, and geographically implausible positions coinciding with known zones of electronic warfare. These inaccuracies hinder efforts to locate breeding colonies, identify key stopover habitats, and assess disease transmission risks posed by migratory birds, particularly for zoonoses such as highly pathogenic avian influenza (HPAI) H5N1. Our findings illustrate how modern conflicts now extend their impact into ecological research infrastructures, calling for systematic correction methods and international coordination to safeguard the robustness of movement ecology studies and One Health models in a geopolitically unstable world.

As geopolitical conflicts intensify worldwide, their consequences are extending into domains not traditionally associated with them, affecting human social, economic and demography dynamics, and increasingly shaping the conditions under which scientific research is conducted (Flack et al. 2022). Conflicts have historically constrained ecological monitoring through restricted access to field sites, damaged research infrastructure, and disrupted international collaboration (Stachowicz et al. 2024). Ecological research has long been intertwined with surveillance and military infrastructures, shaping both the possibilities and the limits of environmental monitoring (e.g., Benson 2010; Blair 2022). Today, however, their influence increasingly extends into the technological systems used to observe and monitor ecological processes.

Animal tracking technologies based on global navigation satellite systems (GNSS) are not neutral observational tools, but are instead supported by political, military, and economic infrastructures embedded in the current geopolitical crisis over which researchers have little control. Modern GNSS-based remote tracking systems rely on constellations operated by multiple state controlled satellite infrastructures—such as GPS (United States), GLONASS (Russia), Galileo (European Union), or BeiDou (China)—whose governance is inherently shaped by national strategic, military, and geopolitical interests. With the exception of the European Galileo system, they are all linked to confrontational national military operations (Larsen 2024), introducing potential vulnerabilities for civilian, commercial and scientific users who depend on their continuous and interference free functioning. Reliable, continuous, and globally accessible GNSS data are essential for disciplines such as movement ecology and One Health research, which depend on large volumes of high-resolution information to track animal movements, monitor disease vectors, and understand ecosystem-level interactions across broad geographic scales (Blair 2022; Lawrence et al. 2025).

Unfortunately, such efforts may now be hampered by growing disruption of GNSS signals in regions experiencing political or military tension, revealing how geopolitical dynamics can directly influence the reliability of globally used satellite-based tracking services (Felux et al. 2025). When modern conflicts involve electronic warfare, availability and reliability of GNSS data is affected, including transportation, communication systems, citizen mobile phones and animal tracking devices involved in ecological research (Westbrook 2019). GNSS receivers are very sensitive to military jamming and spoofing strategies that transmit noise signals across one or more GNSS frequencies to block data tracking (Jiguet et al. 2025). Here we show how such geopolitical dynamics are now directly affecting wildlife tracking data collected in Europe. Using GPS tracking of black-headed gulls (*Chroicocephalus ridibundus*) migrating between southern Europe and breeding areas in Russia and Ukraine (Fig. 1A), we demonstrate that military-induced interference with GNSS signals can generate erratic and geographically implausible positions that compromise the reliability of movement datasets.

**Fig. 1:**
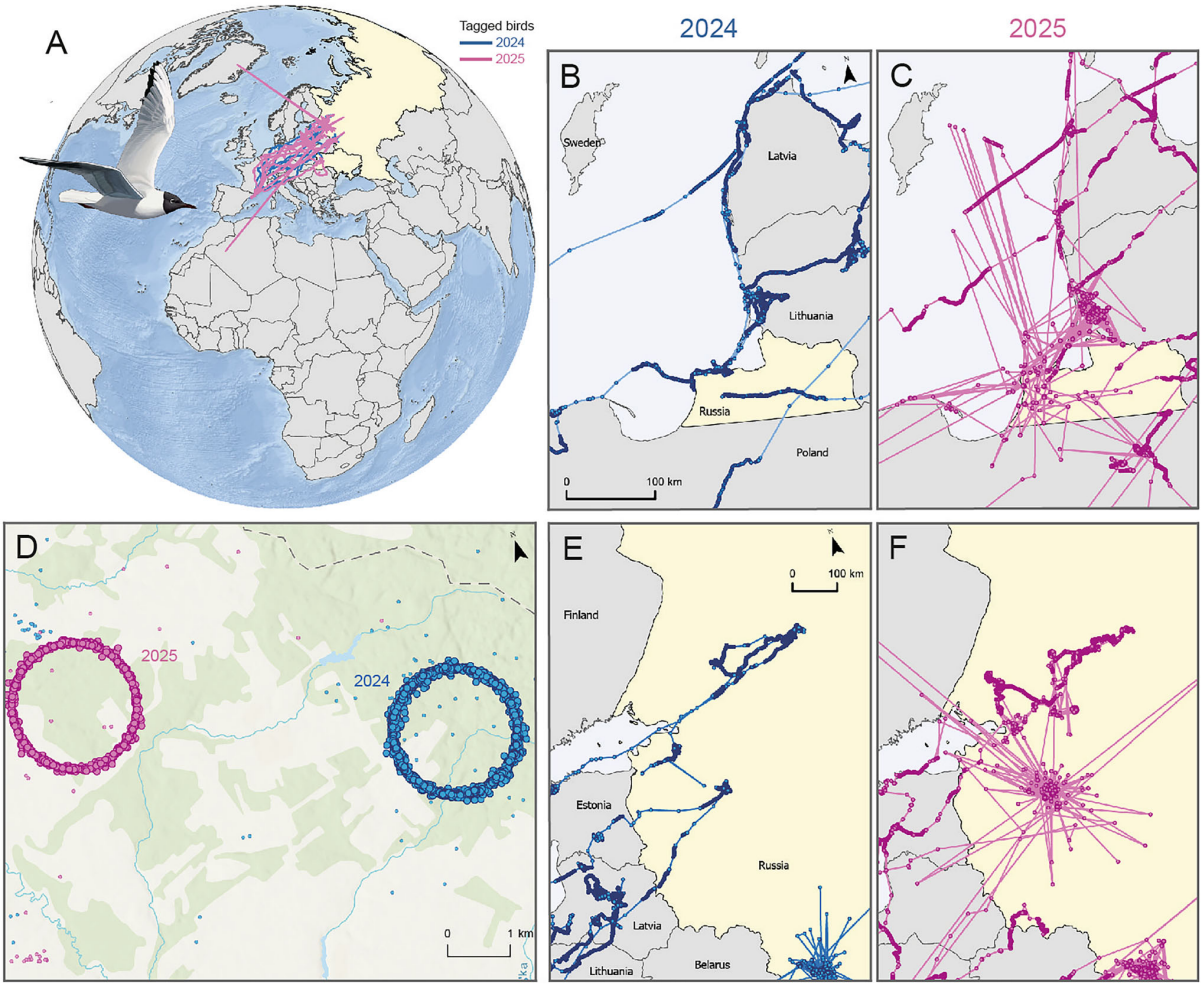
Migration patterns according to uncorrected GPS data of tagged Black-headed gulls in 2024 (blue) and 2025 (pink) from the winter tagging site in Spain towards Russia and Ukraine (in yellow) (**A**). Close-ups: GPS trajectories in Kaliningrad during 2024 (**B**) and 2025 (**C**); erratic circular patterns in Russia (**D**); GPS trajectories in Novgorod during 2024 (**E**) and 2025 (**F**). Gull illustration by Martí Franch

Due to signal interference in or near areas of conflict, movement data showed a higher proportion of GPS positioning errors, in 2024 and 2025, as outliers in Kaliningrad (Fig. 1B and C) and concentric patterns (Fig. 1D). These inaccuracies have escalated in 2025 (Fig. 1), and inhibit the study of migratory movements of GPS tagged gulls (and, no doubt, other tagged birds) in these areas. Some erroneous positions for our GPS tagged gulls fall in countries well beyond the migratory routes of the species (e.g. Algeria or Greenland; see Fig. 1A).

Positions within the Russian boundaries showed a higher proportion of low-quality precision data and erratic patterns (see panels B-F from Fig. 1), making it harder to meet our initial research objectives. For example, identifying the precise location of breeding colonies has important implications for conservation programmes targeting this gull species and, by extension, other waterbirds that breed more successfully within its colonies. Furthermore, migratory wildlife species such as the black-headed gull act as reservoirs for various pathogens, including those of significant public health concern, such as the Highly Pathogenic Avian Influenza (HPAI) H5N1 virus (Plaza et al. 2025) and antibiotic resistant zoonotic bacteria (Martín-Vélez et al. 2024). The inability to study animal movements in areas with unreliable GPS signals hinders the identification of risk-prone habitats such as landfills or wastewater treatment plants, which may act as reservoirs for the dissemination of antibiotic resistant bacteria by gulls (Martín-Vélez et al. 2024). It also limits the development of predictive risk maps as early warning systems for the spread of avian influenza (Plaza et al. 2025). Reliability of predictive models based on animal tracking to prevent disease transmission may therefore be reduced due to current conflicts in Europe. Furthermore, such signal interference can prevent the study of the impacts of conflict on bird movements themselves (Russell et al. 2024).

These findings demonstrate that international conflicts now extend their influence into the reliability of ecological datasets. For animal movement ecology and disease ecology alike, there is an urgent need to acknowledge and correct for GPS disruptions when conducting analyses and building predictive models. Only by integrating awareness of such geopolitical interferences can researchers ensure that conservation and public health decisions are based on robust evidence (Simon et al. 2025). Scientists studying animal movements should also be aware of the possible errors resulting from interference in other areas of conflict, especially in the context of current and future geopolitical tensions.

The ability to observe animal movements is unevenly distributed across space and time, shaped not only by ecology but also by global wealth distribution (Steenbrink et al. 2026), political decisions and security priorities. Recognizing this lack of neutrality is essential for interpreting tracking data, particularly when absences, gaps, or anomalies may reflect geopolitical interferences rather than biological processes. This vulnerability also raises important questions about how ecological research may need to adapt in an increasingly unstable geopolitical context. Rather than representing a straightforward opportunity for technological innovation, growing geopolitical constraints highlight the need for methodological flexibility, including improved error-detection approaches, greater awareness of infrastructure-related biases in datasets, and the continued use of traditional low-tech methods for marking and monitoring (e.g. bird ringing).

Contemporary geopolitical conflicts now extend their influence into the infrastructures that underpin ecological and One Health research. Failure to account for GNSS disruptions may lead to biased movement analyses and weakened predictive models, with consequences for conservation planning and disease risk assessment. There is therefore an urgent need for the movement ecology community to systematically identify, report, and correct for geopolitical sources of error in tracking data. Integrating geopolitical awareness into study design and interpretation will be increasingly necessary to ensure that scientific evidence used to inform conservation and public health decisions remains robust in a rapidly changing world.
